# Primary intracerebral aspergillosis in an immunocompetent patient with no risk factors or clear cause: a case report

**DOI:** 10.1097/MS9.0000000000003861

**Published:** 2025-09-17

**Authors:** Nahar Ismaiel, George Bashour, Isabelle Mhanna, Hayyan Ibrahem, Mohamad Joha, Abbas Hamza, Mohammed Abdulrahman, Zuheir Alshehabi

**Affiliations:** a Department of pathology, Cancer Research Center, Tishreen University Hospital, Latakia, Syria; b Faculty of Medicine, Tishreen University, Latakia, Syria; c Department of Neurology, Tishreen University Hospital, Latakia, Syria; d Department of Neurosurgery, Tishreen University Hospital, Latakia, Syria; e Department of Pathology, Tishreen University Hospital, Latakia, Syria

**Keywords:** case report, immunocompetent patient, intracranial aspergillosis

## Abstract

**Introduction:**

Intracranial aspergillosis (ICA) is an uncommon, life-threatening central nervous system infection that is caused by the *Aspergillus* species. Usually, ICA happens in immunocompromised patients. However, it can sometimes happen in immunocompetent patients, which poses a unique diagnostic and therapeutic challenge. Here we present a rare case of primary ICA in an immunocompetent patient.

**Presentation:**

A 60-year-old woman presented with aphasia, blurry vision, nausea, and vomiting in addition to a 1-month history of eye pain and decreased vision. Examination showed disorientation, gait apraxia, and sphincter dysfunction. A head computed tomography scan showed several hypodense infarction-like lesions, and on magnetic resonance imaging these lesions appeared to be endemic in nature mimicking metastatic lesions. Therefore, a biopsy was taken and subsequent pathological and immunohistochemical examination confirmed the diagnosis of aspergillosis.

**Discussion:**

ICA usually occurs in immunocompromised patients. Nevertheless, it can sometimes happen in immunocompetent patients, and the exact etiology of ICA in immunocompetent patients remains unknown. ICA poses a great diagnostic challenge due to the multiple differential diagnoses that can mimic this condition. Due to this, pathological examination and subsequent IHC staining play a crucial role in diagnosing this condition and differentiating it from other possible diagnoses. Finally, ICA can be treated using antifungal medication and/or surgical resection. However, the prognosis remains poor.

**Conclusion:**

Physicians should keep ICA in mind as it can mimic intracranial lesions (such as metastasis and intracranial tuberculosis), which could be differentiated by either performing a pathological examination on a biopsy or by cerebrospinal fluid polymerase chain reaction tests.

## Introduction

Intracranial aspergillosis (ICA) is a life-threatening fungal infection of the central nervous system (CNS) caused by the *Aspergillus* species^[1]^. While commonly associated with immunocompromised individuals, ICA can also occur in individuals with intact immune systems^[1,2]^. This rare subset of immunocompetent ICA presents a unique diagnostic and therapeutic challenge due to its atypical presentation and potentially worse prognosis than its immunocompromised counterpart^[1,3]^. Symptoms are often non-specific, ranging from simple headaches and altered mental status to neurological deficits and seizures, it also usually presents as a lesion on neuroimaging modalities, which leads to misdiagnosis with tuberculous meningitis, tumor, or abscess^[4,5]^ Here we report a rare case of intracerebral aspergillosis in an immunocompetent patient with no risk factors. This case report has been reported in line with the SCARE criteria^[6]^.

**HIGHLIGHTS**

- Intracranial aspergillosis (ICA) is a life-threatening fungal infection of the central nervous system caused by the *Aspergillus* species.
- ICA can rarely occur in individuals with intact immune systems.
- ICA poses a great diagnostic challenge due to the multiple differential diagnoses that can mimic this condition.
- Treatment for ICA recently is more focused on voriconazole as the first line

## Presentation

A 60-year-old retired female nurse presented to the emergency room at our hospital with aphasia, blurry vision, nausea, and vomiting. Upon interrogation, it was found that her aphasia developed gradually over the past few months and she had a 1-month history of eye pain and decreased vision in addition to that it was noted that the patient’s behavior changed recently. The patient had no significant past medical history of immunosuppression including no diabetes or chronic obstructive pulmonary disease and denied any history of smoking, drug, or any substance abuse.

At examination, the patient was severely disoriented and showed gait apraxia in addition to sphincter dysfunction. Examining her eyes using an ophthalmoscope showed severe bilateral papillary edema. The patient was admitted to the intensive care unit (ICU) for close monitoring.

Laboratory tests were ordered and they showed severe thrombocytopenia (30 × 10^9^/L) and leukocytosis (11 500 WBC/mm^3^) with lymphocytes 38% and neutrophils 59%. All other tests were within the normal range including blood glucose (110 mg/dL) and HB1AC (5.1%). Finally, an HIV antibody test was done and it showed negativity.

**Figure 1.**
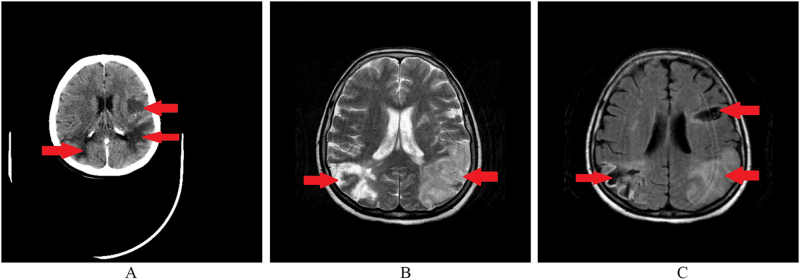
(A) CT scan, axial section. Arrows show several hypodense infarction-like lesions in the cerebral hemispheres. (B) T2 MRI, axial section. Arrows show hyperintense bilateral endemic lesions located in the cerebral hemispheres. (C) FLAIR MRI, axial section. Arrows show hypointense bilateral endemic lesions in the cerebral hemispheres.

A head computed tomography (CT) scan (Fig. 1A) was done, and it showed several hypodense infarction-like lesions located in the left parietal and occipital regions and the right occipital region of the cerebral hemispheres. However, these lesions did not follow a specific arterial pattern. Thus cerebral infarction was ruled out.

A magnetic resonance imaging (MRI) scan with gadolinium showed vast bilateral endemic lesions located in the frontal, temporal, parietal, and occipital regions and oval to round-shaped lesions in the cerebellum. The lesions were hypointense on FLAIR (Fig. 1C) and hyperintense on T2 (Fig. 1B). The differential diagnoses were cerebral metastases and cerebral tuberculosis.

A tuberculin test showed negativity. This was subsequently followed by tuberculosis cultures and interferon-gamma release assays, which were also negative, thus ruling out tuberculosis as a potential cause for the patient’s symptoms.

A surgical biopsy was performed. Under general anesthesia, a right parieto-occipital incision was done followed by adequate dissection until the calvarium was reached. The bone flap was elevated which was followed by a curvilinear durotomy. Tissue samples were sent to pathology for examination. Hemostasis was achieved and the dura was closed. Finally, the bone flap was re-approximated and the wound was closed.

**Figure 2.**
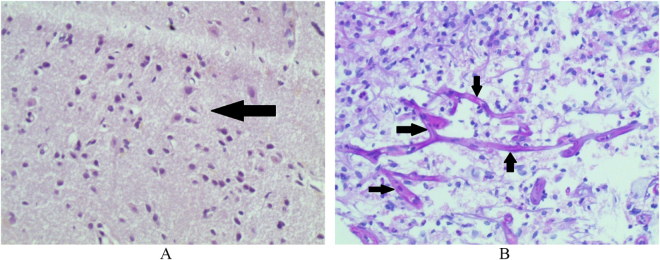
(A) H&E, 100×. Arrow shows severe gliosis. (B) PAS staining. Arrows show broad hyphae angled at 45°.

Pathological examination with hematoxylin & eosin (H&E) stains (Fig. 2A) showed diffuse gliosis suggesting an inflammatory process. Periodic acid–Schiff (PAS) staining (Fig. 2B) showed broad hyphae angled at 45°. These findings confirmed the diagnosis of intracerebral aspergillosis.

After the diagnosis was made, a full-body CT scan was ordered to search for primary fungal lesions that may have caused this intracerebral lesion. However, no fungal lesions were found.

After the surgery, the patient was moved to the ICU for close monitoring and stayed there for 24 h. She was put on intravenous voriconazole (6 mg/kg every 12 h on the first day and 4 mg/kg every 12 h thereafter). After the first 24 h, she was moved to the neurology ward. On the third day after the operation, her vision started improving followed by her aphasia and finally her gait. She remained in the hospital for 90 days at the end of which her disorientation and aphasia subsided, her vision returned to normal and she was able to walk relatively well.

After the completion of the treatment course, an MRI scan was ordered and it showed a drastic reduction in the size of the lesions.

The patient was discharged home with a prescription for voriconazole.

## Discussion

ICA is a rare opportunistic fungal infection of the CNS accounting for 5–10% of all intracranial fungal infections^[2,3]^. However, ICA can sometimes happen in immunocompetent patients, and the exact etiology of immunocompetent ICA remains unclear^[2]^. Primary ICA is only found in a handful of cases in immunocompetent patients.

ICA usually occurs as a result of aspergillus infection of adjacent tissue or through blood transmission, and the most common sites of ICA are the frontal and temporal lobes where it usually presents as a space-occupying lesion^[3,4,7]^.

ICA can present differently depending on the location of the lesion and the most common symptoms include headaches, fever, lethargy, altered mental status, seizures, gait disorders, and focal neurological deficits^[8]^. In immunocompromised patients, ICA can sometimes manifest acutely due to the vascular invasion by *Aspergillus*, which may lead to the formation of thrombi and the eventual development of cerebral infarcts, necrotizing arteritis, mycotic aneurysms, and subarachnoid hemorrhage. This may cause rapid deterioration of the clinical picture usually resulting in death which is in contrast to immunocompetent patients as they often present with a subacute or chronic form of meningitis, cerebral abscess, or solitary granulomas resulting in a better prognosis for these patients^[3–5]^.

Although Radiological findings in ICA are often non-specific, they can be of help in the diagnostic process^[8]^.

ICA is divided radiologically into four general patterns depending on the spreading route of the organism and the immune status of the patient and these are; single or multiple infarcts, single or multiple ring lesions (consistent with abscess formation), solid enhancing lesions (aspergillomas), and finally dural or vascular infiltration from other fungal lesions^[5]^.

ICA appears as iso- or hypointense lesions on T1-weighted MRI and extremely hypointense on T2-weighted images^[3,8]^. After the administration of gadolinium bright enhancement is noted, which is mostly limited to the rim. However, homogenous enhancement can sometimes be seen^[3]^.

ICA poses a great diagnostic challenge due to the multiple differential diagnoses that can mimic this condition. The main differential diagnoses are tuberculomas, meningiomas, metastatic lesions and other space-occupying lesions, septic emboli, and multiple infarcts^[3]^. In our case, the clinical presentation along with the CT suggested a preliminary diagnosis of an infarction, however, the lesion did not follow a specific arterial pattern and the MRI showed round to oval-shaped lesions that are more suggestive of an infection like tuberculosis.

Pathological examination and subsequent PAS staining can differentiate ICA from other entities. The most prominent feature of ICA on H&E stains is a granulomatous reaction with the presence of multinucleated giant cells, neutrophils, plasma cells and eosinophils. PAS stains show 45°-angled or dichotomous branching hyphae which is highly suggestive of aspergillus infection^[5,9]^.

Although ICA is rare, it can have devastating outcomes as the prognosis is very poor and even with new and improved treatments, the mortality rate reached 45.1%^[4]^. Also, the lack of studies can make building a guideline for treatment very challenging as most of these studies are individual case reports that lack the long-term follow-up data necessary to build sufficient guidelines. Treatment for ICA recently is more focused on voriconazole as the first line with improved results over the older Amphotericin B^[4]^. Bora *et al* showed more promising results with combined surgical and medical intervention.^[3]^Although overall mortality was 45.5%, which is close to pure voriconazole treatment, the total resection mortality rate reached as low as 25%^[3]^ In our case, the patient’s lesions were in a sensitive location with no safe ability to be removed surgically in addition to the widespread infiltration of the parenchyma of the frontal, temporal, and occipital lobes; thus, the decision was made to treat the patient using antifungal medications.

## Conclusion

ICA is a rare and deadly fungal infection that is most commonly associated with immunocompromised patients. However, it can rarely happen in immunocompetent individuals with no history of immunosuppression. Here we presented a rare case of ICA in an immunocompetent patient. Although multiple imaging modalities like CT and MRI scans can help diagnose this lesion, diagnosing it remains a challenge as it can mimic many intracranial lesions which could be differentiated by performing a pathological examination on a biopsy. Physicians should keep this diagnosis in mind even in patients who do not have traditional risk factors (such as immunosuppression) as it can happen in immunocompetent patients causing devastating effects on patients and their families.

## Research registration unique identifying number (UIN)

Not required.
